# An oral cancer vaccine using *Bifidobacterium* vector augments combination of anti-PD-1 and anti-CTLA-4 antibodies in mouse renal cell carcinoma model

**DOI:** 10.1038/s41598-023-37234-6

**Published:** 2023-06-20

**Authors:** Hideto Ueki, Koichi Kitagawa, Mako Kato, Shihoko Yanase, Yasuyoshi Okamura, Yukari Bando, Takuto Hara, Tomoaki Terakawa, Junya Furukawa, Yuzo Nakano, Masato Fujisawa, Toshiro Shirakawa

**Affiliations:** 1 Department of Urology, Kobe University Graduate School of Medicine, Kusunoki-cho, Chuo-ku, Kobe, 650-0017 Japan; 2 Laboratory of Translational Research for Biologics, Department of Advanced Medical Science, Kobe University Graduate School of Science, Technology and Innovation, Kusunoki-cho, Chuo-ku, Kobe, 650-0017 Japan

**Keywords:** Cancer, Cancer therapy, Tumour immunology, Urological cancer

## Abstract

Recently, immune checkpoint inhibitor (ICI) based combination therapies, including anti-PD-1 antibody, nivolumab with anti-CTLA-4 antibody, and ipilimumab have become the primary treatment option for metastatic or unresectable renal cell carcinoma (RCC). However, despite the combination of two ICIs, 60–70% of patients are still resistant to first-line cancer immunotherapy. In the present study, undertook combination immunotherapy for RCC using an oral cancer vaccine (*Bifidobacterium longum* displaying WT1 tumor associated antigen (*B. longum* 420)) with anti-PD-1 and anti-CTLA-4 antibodies in a mouse syngeneic model of RCC to explore possible synergistic effects. We found that *B. longum* 420 significantly improved the survival of mice bearing RCC tumors treated by anti-PD-1 and anti-CTLA-4 antibodies compared to the mice treated by the antibodies alone. This result suggests that *B. longum* 420 oral cancer vaccine as an adjunct to ICIs could provide a novel treatment option for RCC patients. Our microbiome analysis revealed that the proportion of *Lactobacilli* was significantly increased by *B. longum* 420. Although the detailed mechanism of action is unknown, it is possible that microbiome alteration by *B. longum* 420 enhances the efficacy of the ICIs.

## Introduction

In recent years, two immune checkpoint inhibitors (ICIs) have been approved as standard of care for metastatic or unresectable renal cell carcinoma (RCC). Nivolumab, an anti-PD-1 antibody, was approved in Japan in 2016^1^, and ICI-based combination therapies, such as the anti-PD-1 antibody nivolumab with the anti-CTLA-4 antibody ipilimumab, have become the primary treatment option for metastatic RCC since 2018^2^. However, despite the combination of two ICIs, 60–70% of patients are resistant to primary ICI treatment and many patients still do not fully benefit from ICI^3^.

The fundamental principle of cancer immunotherapy, including ICI, is to activate tumor-specific cellular immunity. Many studies have been conducted on cancer vaccines that can artificially induce tumor immunity and can be used in combination with ICI^4,5^. Cancer vaccines, which can forcibly induce tumor-specific cellular immunity through antigen presentation by dendritic cells, are a promising treatment strategy for enhancing the efficacy of cancer immunotherapy^5,6^. The National Cancer Institute has ranked tumor associated antigens (TAA) as targets for cancer vaccines based on criteria such as therapeutic efficacy, immunogenicity, carcinogenicity, specificity, expression level and positive cell rate. In this ranking, Wilms' tumor (WT) 1 protein ranked first in their TAA Ranking for Potential Application in Cancer Vaccine Therapy^7^. The WT1 has been reported to be overexpressed in a variety of tumors, including leukemia, breast cancer, and pediatric kidney tumors (Wilms' tumor) and to play an oncogenic role in other cancer types including renal and bladder cancer^8–10^.

Although many clinical trials of WT1 cancer vaccines using WT1 short peptides have been performed, the results of these clinical trials were disappointing^11,12^. It is obvious that technological innovations, such as the development of novel antigen delivery systems, are needed to develop more effective cancer vaccines. We previously developed an oral vaccine platform using *Bifidobacterium longum* (*B. longum*) as the vector for antigen protein delivery to the intestinal immune system^6^. We then constructed an oral cancer vaccine using recombinant *B. longum* expressing the greatest length of WT1 protein on the cell surface (*B. longum* 420)^13^ and demonstrated that *B. longum* 420 could induce multiple WT1 epitope-specific cellular immunity and significantly inhibit tumor growth in WT-1-expressing mouse prostate cancer and bladder cancer models^4,14^. *B. longum*, an endemic intestinal bacterium, is recognized as an ideal vector for an oral vaccine platform because it strongly adheres to human intestinal epithelial cells and induces activation of dendritic cells in Peyer's patches (PP)^15^. Furthermore, oral administration of *B. longum* has been demonstrated in melanoma to enhance the efficacy of immunotherapy by changing the intestinal microbiota^16^.

To date, our laboratory has successfully induced strong cellular immunity in mouse models of bladder and prostate cancer with this oral vaccine^4,14^. However, it is not clear whether oral administration of *B. longum* 420 has a therapeutic enhancing effect in renal cell carcinoma, for which ICIs are the mainstay of treatment. The question in this study was whether *B. longum* 420 exerts an anti-tumor effect on renal cell carcinoma through immune induction and by alteration of the intestinal microbiota. We evaluated cellular immunity and alteration of microbiota induced by *B. longum* 420 and the synergistic effect of combining *B. longum* 420 with anti–PD-1 and anti-CTLA-4 antibodies in an RCC syngeneic mouse tumor model.

## Results

### B. longum 420 and anti-PD-1 antibody suppressed Renca tumor growth

#### Tumor volume

**Figure 1:**
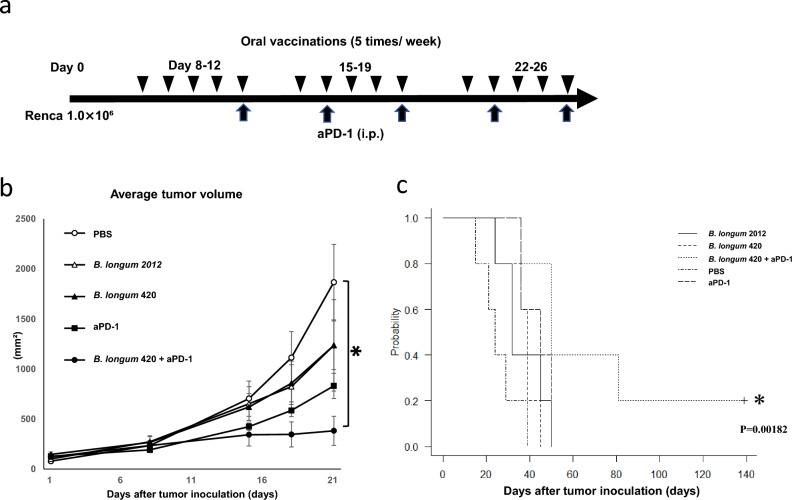
Antitumor effect of *B. longum* 420 oral administrations and anti-PD-1 antibody against Renca. (**a**) Treatment schedule for oral administration of *B. longum* 420 with anti-PD-1 antibody Mice (n = 5) were orally vaccinated with *B. longum* 420, *B. longum* 2012, or PBS 5 times a week for 4 weeks after the tumor inoculation with Renca cells. Anti-PD-1 antibody or saline control was intraperitoneally injected into mice at days 12, 16, 19, 23, and 26 after tumor inoculation. (**b**) Average tumor growth curves Average tumor growth curves are shown: PBS, *B. longum* 2012, *B. longum* 420 alone, PBS + anti-PD-1 antibody alone, and *B. longum* 420 with anti-PD-1 antibody. *B. longum* 420 with anti-PD-1 antibody significantly suppressed tumor growth compared to the PBS group at day 21 (*p < 0.05). Each data point presents the average tumor volumes of each group (bars, ± SE). (**c**) Kaplan–Meier survival curve Combination therapy significantly improved the survival rate compared to the other treatment groups (*p < 0.05).

The *B. longum* 2012 strain with a plasmid carrying galacto-N-biose/lacto-N-biose I binding protein (GL-BP) gene only, constructed in our previous study, was used as a control^6^. GL-BP is a membrane protein in the ATP-binding cassette transporter on the wild-type *B. longum* cell wall, which we used as an anchor to display antigen on the bacterial cell surface. Oral administration of *B. longum* 420 combined with intraperitoneal injection of mouse anti-PD-1 antibody significantly suppressed the growth of Renca tumors compared to the PBS group at day 21 (*p* < 0.05) (Fig. 1a,b). In the other groups (n = 5), all mice died from tumor growth, with an average survival of 25.6 ± 5.8 (standard error) days with PBS control, 36.6 ± 7.6 days with *B. longum* 2012, 36.6 ± 5.7 with *B. longum* 420, and 43.4 ± 5.3 days with anti-PD-1 antibody alone. The survival curve for the combination of *B. longum* 420 with anti-PD-1 anti-body treatment showed a significant prolongation of survival of 71.2±29.9 days compared with the other treatment groups (Fig. 1c, *p* < 0.05).

#### TILs

**Figure 2:**
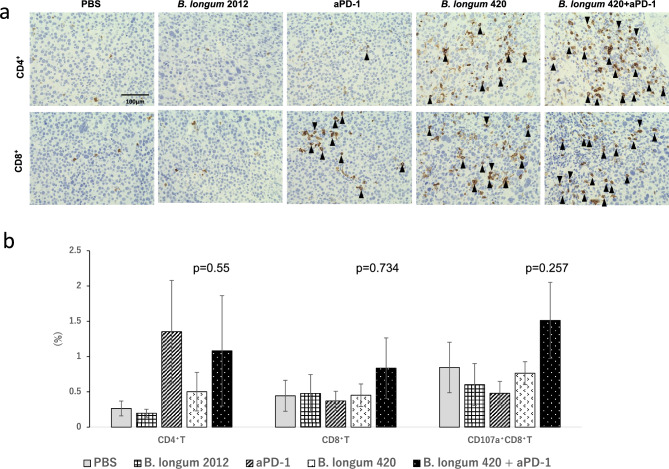
Evaluation of the tumor infiltrating lymphocyte in experiment using anti-PD-1 antibody for immune checkpoint inhibitor. (**a**) Immunohistochemical staining for tumor-infiltrating T cells in Renca tumors. Immunohistochemical staining showed remarkably increased numbers of CD4 and CD8 T cells in tumor tissues in mice treated with combination therapy. (**b**) Tumor-infiltrating T cells after treatment in Renca model The population of tumor-infiltrating CD4^+^T, CD8^+^T, and CD107a^+^ CD8^+^T cells was analyzed by flow cytometry (n = 5). Each data point represents the average of the cell frequencies (bars, ± SE).

We next collected tumor tissues from another set of mouse treatment groups after treatment to investigate the tumor infiltrating lymphocytes (TILs). Immunohistochemical staining for CD4^+^ and CD8^+^ T cells showed remarkably increased numbers of CD4^+^ and CD8^+^ T cells infiltrating into the tumor tissues in mice treated with the combination of *B. longum* 420 and anti-PD-1 antibody compared to the other treatment groups (Fig. 2a). The flow cytometric analysis of T cells isolated from tumor tissues revealed that the combination of *B. longum* 420 and anti-PD-1 antibody treatment increased tumor-infiltrating CD8^+^, and CD107a^+^CD8^+^ T cells (*p* = 0.734, and 0.257, respectively), while anti-PD-1 antibody alone and combination with *B. longum* 420 increased CD4^+^ T cells compared to the other treatment groups (*p* = 0.55, Fig. 2b), even though there was not significant difference.

### B. longum 420 enhanced the anti-tumor activity of combined anti-PD-1 and anti-CTLA-4 antibodies

#### Tumor volume

**Figure 3:**
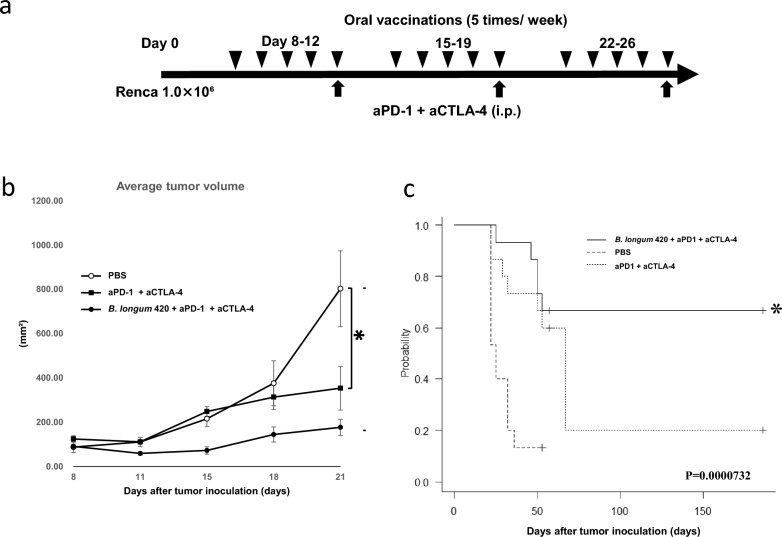
Antitumor effect of *B. longum* 420 oral administrations and anti-PD-1 and anti-CTLA-4 antibody against Renca tumors. (**a**) Treatment schedule for oral administration of *B. longum* 420 with anti-PD-1 and anti-CTLA-4 antibody Mice (n = 15) were orally vaccinated with *B. longum* 420 or PBS 5 times a week for 4 weeks after tumor inoculation with Renca cells. Anti-PD-1 + anti-CTLA-4 antibody or saline control was intraperitoneally injected into mice at days 12, 16, 19, 23, and 26 after tumor inoculation. (**b**) Average tumor growth curves Average tumor volumes from the treatment groups: PBS, PBS + anti-PD-1 + anti-CTLA-4 antibody alone, and *B. longum* 420 with anti-PD-1 + anti-CTLA-4 antibody. *B. longum* 420 with anti-PD-1 + anti-CTLA-4 antibody significantly suppressed tumor growth compared to the PBS group at day 21 (*p < 0.05). Each data point presents the average tumor volumes of each group (bars, ± SE). (**c**) Kaplan–Meier survival curve The combination therapy significantly improved the survival rate compared to the other treatment groups (*p < 0.05).

To confirm the synergistic anti-tumor activity of adding the anti-CTLA-4 antibody to the combination of anti-PD-1 antibody and *B. longum* 420, we next performed further animal studies using the Renca-bearing BALB/c mouse RCC syngeneic tumor model. The combined anti-PD-1 and anti-CTLA-4 antibodies group and the combined oral administration of *B. longum* 420 with anti-PD-1 and anti-CTLA-4 antibodies group both showed significantly suppressed tumor growth compared to the PBS group at day 21 (*p* < 0.05) (Fig. 3b). The survival curve (Fig. 3c) for the combination of *B. longum* 420 with anti-PD-1 and anti-CTLA-4 antibodies showed a significant prolongation of survival of 78.7 ± 14.5 days compared with the other treatment groups (*p* < 0.05). In the other groups, an average survival of 29.5 ± 2.8 days with PBS control, 58.0 ± 9.9 days with anti-PD-1 and anti-CTLA-4 antibody alone.

#### TIL

**Figure 4:**
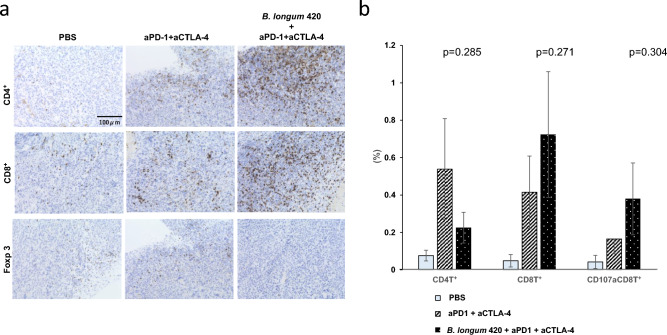
Evaluation of the tumor infiltrating lymphocyte in experiment using anti-PD-1 and anti-CTLA-4 antibody for immune checkpoint inhibitor. (**a**) Immunohistochemical staining for tumor-infiltrating T cells in Renca tumors. Resected Renca tumors were immunohistochemically stained with anti-CD4 antibody, anti-CD8 antibody, or Foxp3 antibody. Representative immunohistochemical staining in each treatment group is shown (400 ×). (**b**) Tumor-infiltrating T cells after treatment in the Renca model. The population of tumor-infiltrating CD4T, CD8T, and CD107a^+^ CD8^+^T cells was analyzed by flow cytometry (n = 5). Each data point represents the average of the cell frequencies (bars, ± SE).

Immunohistochemical staining for CD4^+^ and CD8^+^ T cells showed remarkably increased numbers of CD4^+^ and CD8^+^T cells infiltrating into the tumor tissues in mice treated with the combination of *B. longum* 420 and anti-PD-1 and anti-CTLA-4 antibodies compared to the other treatment groups (Fig. 4a). Interestingly, the anti-PD-1 and anti-CTLA-4 antibodies alone showed the greatest increase in Foxp3+ T cells compared to the other groups (Fig. 4a). The flow cytometric analysis of T cells isolated from the tumor tissues revealed that the combination of *B. longum* 420, anti-PD-1 and anti-CTLA-4 antibodies showed the highest numbers of tumor-infiltrating CD8^+^ and CD107a^+^CD8^+^T cells (*p* = 0.271, and 0.304, respectively), while the anti-PD-1 and anti-CTLA-4 antibodies alone group showed the highest number of CD4^+^ T cells among all the treatment groups **(p** = 0.285, Fig. 4b). Representative dot plots of each treatment group with gating for staining were shown in Supplementary Fig. S1.

### Differences in bacterial community profiles before and after administration in each group

**Figure 5:**
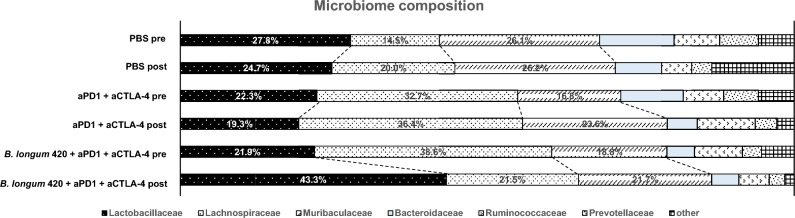
Alterations in gut microbiome composition before and after treatment. The microbiome was analyzed in the following five groups; PBS, PBS with anti-PD-1 + anti-CTLA-4 antibody, and *B. longum* 420 with anti-PD-1 + anti-CTLA-4 antibody. Only the *B. longum* 420 with anti-PD-1 + anti-CTLA-4 antibody group showed an increased proportion of *Lactobacillaceae* after treatment.

Figure 5 summarizes the differences in bacterial microbiota in the PBS, anti-PD-1 and anti-CTLA-4 antibodies, and *B. longum* 420 with anti-PD-1 and anti-CTLA-4 antibodies groups. Although the pre-treatment stool samples of the *B. longum* 420 with anti-PD-1 and anti-CTLA-4 antibodies group showed the lowest proportion (21.9%) of *Lactobacillaceae* at the family level among all treatment groups, after the treatments the *B. longum* 420 and anti-PD-1 and anti-CTLA-4 antibodies group showed the highest proportion (43.3%) of *Lactobacillaceae.* Among all the stool samples tested, the *B. longum* 420 sample showed the highest proportion of *Lactobacillaceae* (43.3% vs 27.8%, 24.7%, 22.3%, 19.3%, and 21.9%).

### Intracellular cytokine staining for splenocytes

**Figure 6:**
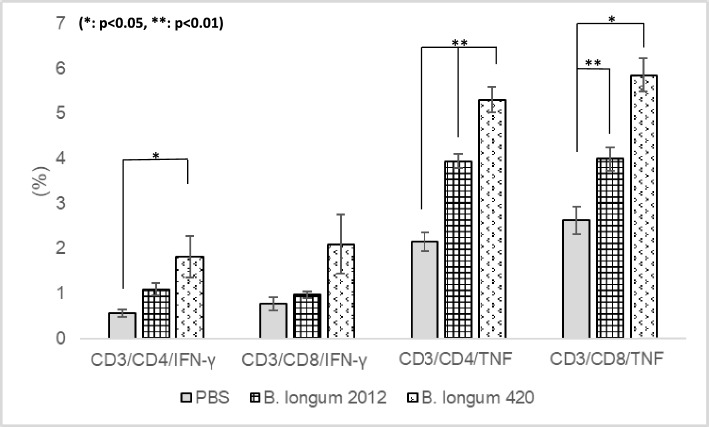
Frequency of cytokine-producing T cells in spleen after oral vaccination of *B. longum* 420. The frequency of IFN-γ and TNF-α-producing T cells were determined by using intracellular cytokine staining after oral vaccination of *B. longum* 420. The oral vaccination of *B. longum* 420 induced significantly higher proportion of CD4T and CD8T cells that were WT1-specifically producing IFN- γ and TNF- α compared with other groups.

To determine the frequency of WT1-specific T cells induced by the oral vaccination of *B. longum* 420, we performed intracellular cytokine staining for splenocytes stimulated with mitomycin-C-treated Renca. The frequency of CD4 + T cells that were WT1-specifically producing interferon (IFN)-γ in th*e B. longum* 420 treated mice group was significantly higher than in PBS group (Fig. 6, *p* < 0.05). Also, the frequency of TNF-α producing CD4 + T and CD8 + T cells in the *B. longum* 420 treated mice group was significantly higher than the other groups (Fig. 6, *p* < 0.05, *p* < 0.01).

## Discussion

The present study resulted in two critical observations. First, *B. longum* 420 significantly augmented the anti-tumor activity in mice loaded with Renca, mouse RCC tumors treated with an anti-PD-1 antibody, and mice treated with anti-PD-1 and anti-CTLA-4 antibodies. Second, the administration of *B. longum* 420 substantially changed the microbiome, and particularly increased the proportion of the *Lactobacillaceae* family.

In our results, the group treated with a combination of anti-PD-1 antibody and *B. longum* 420 had significantly lower average tumor volumes compared to the PBS group (Fig. 1b and significantly better survival compared to the other groups (Fig. 1c). Consistent with these results, the anti-PD-1 and anti-CTLA4 antibodies alone showed a significant tumor growth inhibitory effect compared to the PBS group, and the addition of *B. longum* 420 to these antibodies significantly enhanced the antitumor activity (Fig. 3b). Combination therapy with anti-PD-1 and anti-CTLA4 antibodies and *B. longum* 420 demonstrated a significant improvement in survival compared to the PBS group and the anti-PD-1 and anti-CTLA4 antibodies alone groups (Fig. 3c). These results suggested that even though *B. longum* 420 alone did not show significant antitumor activity, *B. longum* 420 could significantly augment the antitumor activity of ICI treatments in the mouse RCC model. In the clinical setting, the combination of the PD-1 inhibitor nivolumab with the CTLA-4 inhibitor ipilimumab is currently the only dual ICI combination approved in the first-line setting for patients with mRCC^17^. The clinical data show a reduction in risk of death by 35%, impressive long-term PFS (Progression Free Survival) plateauing at approximately 35% after 30 months, and superior quality of life versus sunitinib, making this combination attractive. Although it is highly likely that nivolumab plus ipilimumab has the highest CRR (Clinical Response Rate)^17^, most patients still either have primary resistance to these therapies or acquire resistance after an initial response^2^. Therefore, developing novel therapeutic strategies designed to overcome these resistance mechanisms is of paramount importance for patients with this disease. In this regard, the results of the present study show potential for improving ICI-ICI treatment outcomes further.

Our immunohistochemical study showed that administration of *B. longum* 420 increased the infiltration of both CD4^+^ T cells and CD8^+^ T cells into the tumor tissues (Figs. 2a, 4a). Although there was no statistically significant difference, flow cytometric analysis also showed that the combination of *B. longum* 420 and ICIs (anti-PD-1 anti-body w/wo anti-CTLA-4 antibody) increased the infiltration of CD8^+^ T cells and CD107a^+^ CD8^+^T cells, which are considered activated CD8^+^ T cells, into the tumor tissues (Figs. 2b, 4b). It is well known that CD8^+^T cells play a critical role in the antitumor immune response, and these TIL findings strongly support the adjuvant effect of *B. longum* 420 with ICI treatments in RCC. Interestingly, the flow cytometric analysis showed that the numbers of CD4^+^ T cells in the ICI-alone groups were higher than the combination of *B. longum* 420 and ICIs (Figs. 2b, 4b). The detailed mechanism for this finding is unknown, but there have been several clinical reports that CD4^+^ Foxp3^+^ T cells are increased by ICIs in ICI-resistant tumors^18,19^. Tumor-infiltrating Tregs may promote tumor progression by suppressing the natural anti-cancer immune responses. The percentage of Tregs among CD4^+^ cells is significantly higher in tumors than in the immune organs^20–22^. The increased percentage of Tregs in many cancers is often associated with poor prognosis^23^. Therefore, the impact of Tregs should be considered for successful ICI therapies. Our immunohistochemical study showed that the number of Foxp3^+^ T cells was increased in the anti-PD-1 and anti-CTLA-4 antibodies alone group compared to the other groups (Fig. 4a). We also proved that oral administration of *B. longum* 420 induced WT1- specific CD4 + T and CD8 + T cells in BALB/c mice, by performing intracellular cytokine staining for splenocytes after vaccination. Our previous study using anti-PD-1 antibody in a poorly responsive MBT-2 mouse bladder cancer model revealed that *B. longum* 420 alone induced higher anti-tumor activity compared to the combination of anti-PD-1 antibody and *B. longum* 420. In that study, the anti-PD-1 antibody remarkably increased the number of regulatory T cells^23–25^.

Another interesting finding in this study is the modification of the intestinal microbiome by oral administration of *B. longum* 420. The proportion of *Lactobacilli* was significantly altered after treatment with ICI and *B. longum* 420 (Fig. 5). Since only one mouse in each group was analyzed, we must carefully interpret the results. However, this is an important finding because there have been no studies comparing gut microbiomes before and after the administration of ICI and probiotics including *B. longum*. The host microbiome is established as one of the critical parameters to improve or impair the efficacy of ICI therapies^24–26^. Increasing evidence indicates that some commensal bacteria modulate the tumor microbiome^27^ and that intestinal commensal bacteria-induced IFN γ-producing CD8^+^ T cells augment anti-tumor responses in ICI^28^. For example, Kawanabe-Matsuda et al. reported that microbial exopolysaccharide produced by *Lactobacillus delbrueckii* subsp. bulgaricus OLL1073R-1 (EPS-R1) induced CCR6^+^ CD8^+^ T cells in mice and humans^29^. In mice, ingestion of EPS-R1 augmented the antitumor effects of anti–CTLA4 or anti–PD-1 monoclonal antibody against CCL20-expressing tumors. Although it is unclear whether the mechanism by which *lactobacilli* increase response to ICI is microbiota change or metabolite, there have been a number of reports in recent years that *lactobacillus species* can enhance reactivity to ICI^30,31^。Moreover, it was recently reported that *Bifidobacterium pseudolongum* produced inosine-activated Th1 cells with anti-tumor effects in the presence of IFN-γ or other costimulation^31^. Previous reports have shown that repeated oral administration of enterobacteria alters the intestinal microbiota^32^, for instance the intense efficacy of *B. longum* BB536 (BB536Y group) in improving the intestinal environment, reducing harmful bacteria and improving defecation frequency and stool characteristics in humans^33–35^. Although the exact mechanism and the specific microbiota members associated with the enhanced clinical response of ICIs remains unknown, it is possible that microbiome alteration also contributed to antitumor activities, and the benefits of *B. longum* 420 as a probiotic-based cancer vaccine may provide a novel treatment option for RCC patients resistant to ICIs.

## Methods

### Recombinant Bifidobacterium

Genetically modified recombinant Bifidobacterium, *B. longum* 420 and *B. longum* 2012, were previously constructed^33^. A partial murine-WT1 gene (aa117–419) was synthesized by GensScript (NJ, USA). This synthesized gene segment includes three well-known CD8 + T cell epitopes (126–134, 187–195, and 235–243 amino acids), as well as one established CD4 + T cell epitope (332–347 amino acids) that have already been tested for their immunogenicity in both human and murine models^36–38^. The synthesized WT1 gene was fused to GL-BP, and then corresponding gene was ligated with the *B. longum. B. longum* 2012 expresses only GL-BP and is used as a control agent^34^. All three recombinant Bifidobacterium bacteria were anaerobically cultured in Gifu anaerobic medium (Nissui, Tokyo, Japan) with 50 mg/mL spectinomycin at 37 °C. After cultivation, these recombinant bacteria were heated for inactivation at 65 °C for 5 min.

### Cell line

Renca**,** a renal cell carcinoma cell line expressing murine-WT1 protein derived from BALB/c, was purchased from ATCC and maintained in in Eagle’s minimum essential medium supplemented with 10% fetal bovine serum (Sigma-Aldrich Japan, Tokyo, Japan) and 1% penicillin–streptomycin (Nacalai Tesque, Kyoto, Japan). The overexpression of WT1 protein in Renca was confirmed by western blotting analysis (data not shown).

### Oral vaccination

Dose of oral vaccination and treatment schedules were determined based on our previous studies^4,5,13^. Male BALB/c mice were orally given 100 μL of PBS, 1.0 × 10^9^ CFU of *B. longum* 420, or *B. longum* 2012 5 times a week for 4 weeks (days 7–11, 14–18, 21–25, and 28–32) with a feeding needle. After 3 weeks of vaccination, mice were euthanized and tumors were resected to investigate the local immune response.

### Animal experiments for combination therapy

To explore the in vivo anti-tumor activity of the combination of *B. longum* 420 and anti-PD-1 antibody against renal cell carcinoma, we employed a Renca mouse RCC syngeneic subcutaneous tumor model. One million Renca cells were subcutaneously injected into male BALB/c mice at day 0. A total of 25 mice with Renca subcutaneous tumor were randomly assigned to 5 treatment groups (n = 5): *B. longum* 420 + anti-PD-1, *B. longum* 420, *B. longum* 2012, PBS + anti-PD-1, and PBS at day 7, and then oral administrations were carried out as described above (Fig. 1a). Anti-mouse PD-1 antibody (RMP1-14-derived mouse monoclonal antibody against murine PD-1, InvivoGen, San Diego, CA, USA) was used for the anti-PD-1 treatment, and saline was used as an isotype control. Two hundred milligrams of anti-PD-1 antibody or isotype control was intraperitoneally injected into mice at days 12, 16, 19, 23, and 26. Tumor volume was measured by the calculation formula of (longest diameter) × (shortest diameter)^2^ × 0.5. Mice were euthanized when their tumors grew larger than 20 mm in diameter, and Kaplan–Meier survival curves were generated.

Next, we prepared another set of mice to examine the in vivo anti-tumor activity of the combination of *B. longum* 420, anti-PD-1 antibody and anti-CTLA-4 antibody against renal cell carcinoma. A total of 45 mice with Renca subcutaneous tumor were randomly assigned to 3 treatment groups (n = 15): *B. longum* 420 + anti-PD-1 + anti-CTLA-4, PBS + anti-PD-1 + anti-CTLA-4, and PBS at day 7, and then oral administrations were carried out as in the previous study (Fig. 3a). Anti-mouse CTLA-4 antibody (9D9-derived mouse monoclonal anti-body against murine CTLA-4, InvivoGen, San Diego, CA, USA) was used for the anti-PD-1 + anti-CTLA4 treatment. 2.5 μg of anti-PD-1 antibody and anti-CTLA-4 were intraperitoneally injected into mice at days 12, 19, and 26.

### Immunohistochemical study

Another set of mice was injected with 1.0 × 10^6^ Renca and treated by the same method described above. Tumors were resected and divided into two pieces, and half were fixed with 4% paraformaldehyde-PBS and embedded in paraffin. Another piece of tumor was used for flow cytometry as described below. Paraffin embedded tumor tissue sections were deparaffinized and rehydrated. Antigen retrieval was performed in Bond epitope retrieval buffer (pH6.0 for CD8, pH9.0 for CD4; Leica Microsystems, Wetzlar, Germany) at 98C for 20 min. Immunohistochemical staining was performed in an automatic tissue processor (Leica Microsystems Bond) according to the manufacturer's standard protocol. Briefly, tissue sections were incubated at RT for 15 min with rabbit anti-mouse CD4 antibody (1:1,000, Abcam) or rabbit anti-mouse CD8 antibody (1:400, Cell Signaling Technology Japan, Tokyo, Japan). After washing, sections were incubated with horseradish peroxidase-conjugated secondary antibodies. After washing, sections were incubated with 3,30 diaminobenzidine and counterstained with hematoxylin. Anti-mouse CD4 antibody (1:1,000, Abcam, Cambridge, UK), anti-mouse CD8 antibody (1:400, Cell Signaling Technology Japan, Tokyo, Japan), and Foxp3 antibody 236A/E7 (Mouse monoclonal anti-body; Abcam, Cambridge, UK) were used in the immunohistochemical staining. The tissue slides were observed with a BZ-X710 microscope (Keyence, Osaka, Japan). Evaluation of immunostaining was performed by two of the authors independently (Ueki and Kitagawa). Target areas were selected for each specimen using free-form drawings, and the tissue was evaluated under 20-fold magnification. For regular sections, whole areas were screened under 20-fold magnification, and three representative high-power fields (HPF) were assessed for cell counts in every specimen (400-fold magnification).

### Comparison of the gut microbiota composition

To observe whether *B. longum* 420 administration caused changes in the intestinal microbiota, fecal samples were collected before (day 7) and after (day 22) *B. longum* 420 administration. From each group, n = 1 was selected for microbiome analysis (TechnoSuruga Laboratory Co, Shizuoka, Japan). The mouse with the largest tumor size in the PBS and ICI monotherapy group was selected, and the mouse with the smallest tumor size in the *B. longum* 420 combination group was selected. DNA extraction was conducted according to a previously described method^39^ using an automated DNA isolation system (GENE PREP STAR PI-480 KURABO, Japan). The V3-V4 regions of Bacterial and Archaeal 16S rRNA were amplified using the Pro341F/Pro805R primers and dual-index method^39,40^. Barcoded amplicons were paired-end sequenced on a 2 × 301-bp cycle using the MiSeq system with MiSeq Reagent Kit version 3 (600 Cycle). The primer sequences on paired-end sequencing reads were trimmed by Cutadapt ver 1.18 with default settings^41^. Paired-end sequencing reads were merged using the fastq-join program with default settings^42^. Only joined-reads that had a quality value score of ≥ 20 for more than 99% of the sequence were extracted using the FASTX-Toolkit^43^. The chimeric sequences were deleted with usearch61^44,45^. Nonchimeric reads were submitted for 16S rDNA-based taxonomic analysis using the Ribosomal Database Project ver 2.13 (RDP) and the TechnoSuruga Lab Microbial Identification database ver 16.0 (DB-BA, TechnoSuruga Laboratory, Japan) with homology for ≥ 97%^46,47^.

### Intracellular cytokine staining for splenocytes

Another male BALB/c mice were orally given 100 μL of PBS, 1.0 × 10^9^ CFU of *B. longum* 420, or *B. longum* 2012 5 times a week for 4 weeks (days 0–4, 7–11, 14–18, and 21–25) as described above. After the last oral vaccination, spleens were resected and single cell suspensions were collected. The splenocytes (2.0 × 10^6^) were cultured and re-stimulated with 2.0 × 10^5^ mitomycin-C-treated Renca cells in vitro. GolgiStop (BD Biosciences, San Jose, CA) was added to the medium after 26 h of the cell cultivation, and then the cells were cultured for additional 12 h. The cells were collected and processed using a BD Cytofix/CytopermTM Plus Fixation/Permeabilization Kit (BD Biosciences) for intracellular cytokine staining assay according to our previous studies^4,14^. As for the intracellular staining, PE-anti-IFN- γ or PE-anti-TNF- α (BD Biosciences, respectively) were used in this study. The stained cells were counted and analyzed by using Guava flow cytometer (Luminex, Austin, TX).

### Statistical analysis

One-way ANOVA followed by the Tukey–Kramer method was employed for the comparisons between multiple groups. The log-rank test on Kaplan–Meier curves was employed for the statistical analysis of survival between groups. Differences among experimental groups were considered significant when *p* < 0.05.

### IRB

All experiments and methods were performed in accordance with the relevant guidelines and regulations, and all experimental protocols, including animal experimental designs and procedures, were reviewed and approved by the institutional ethics and animal welfare committees of the Kobe University Graduate School of Medicine.

## Supplementary Information

Supplementary Information.

## Data Availability

The datasets generated and/or analyzed during the current study are available from the corresponding author on reasonable request.
