# Supplementary material for: An oral cancer vaccine using Bifidobacterium vector augments combination of anti-PD-1 and anti-CTLA-4 antibodies in mouse renal cell carcinoma model
Source: Sci Rep. 2023 Jun 20;13:9994. doi: 10.1038/s41598-023-37234-6 (PMC10281997; doi:10.1038/s41598-023-37234-6)
Supplement: Supplementary file 1 — Supplementary Information. [file 41598_2023_37234_MOESM1_ESM.pptx]

## Slide 1
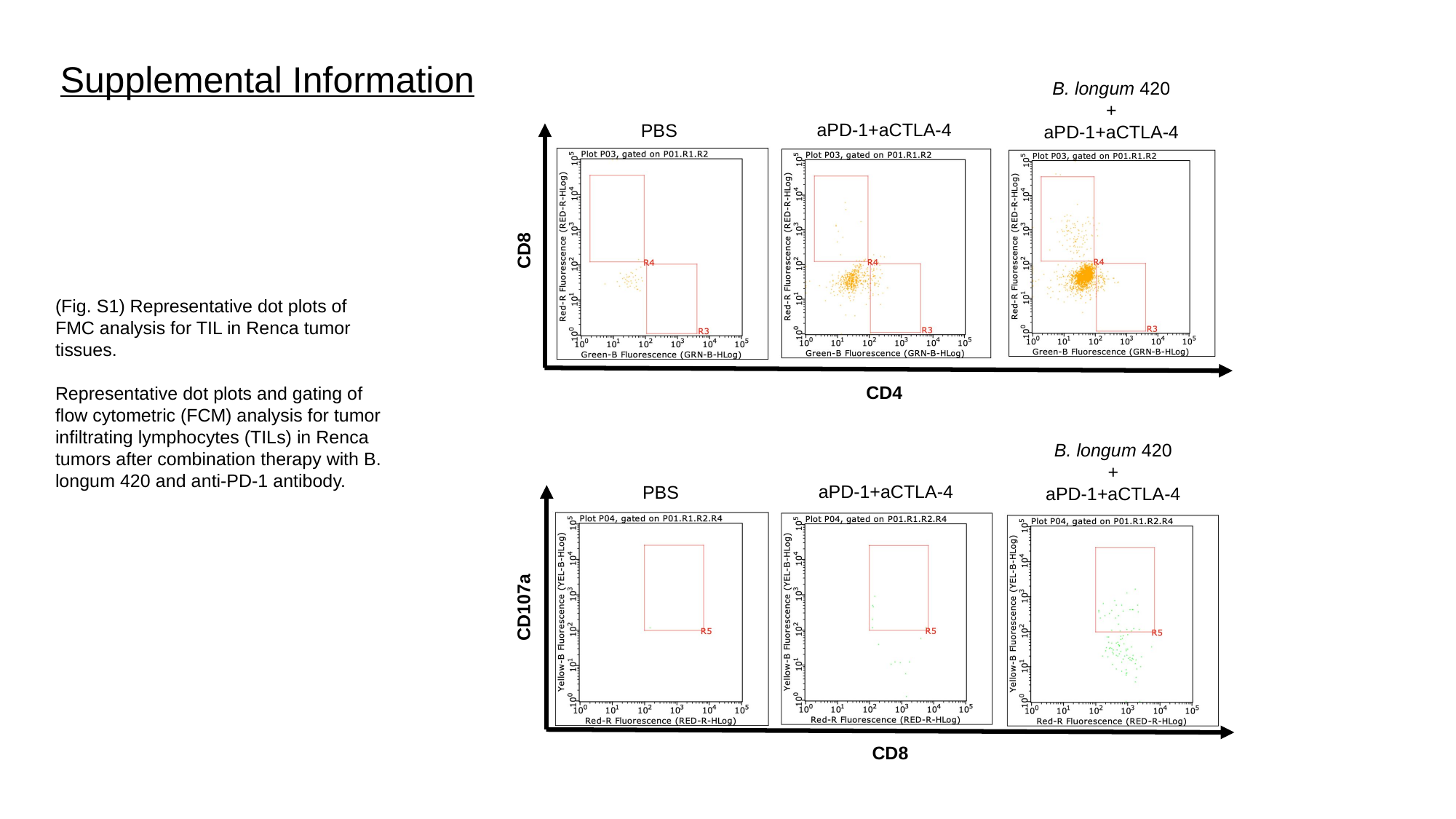

# Supplemental Information
B. longum 420
+
aPD-1+aCTLA-4
aPD-1+aCTLA-4
PBS
CD8
(Fig. S1) Representative dot plots of FMC analysis for TIL in Renca tumor tissues.
Representative dot plots and gating of flow cytometric (FCM) analysis for tumor infiltrating lymphocytes (TILs) in Renca tumors after combination therapy with B. longum 420 and anti-PD-1 antibody.
CD4
B. longum 420
+
aPD-1+aCTLA-4
aPD-1+aCTLA-4
PBS
CD107a
CD8
